# Why is greater medication adherence associated with better outcomes

**DOI:** 10.1186/1742-7622-10-1

**Published:** 2013-02-02

**Authors:** Arthur Hartz, Tao He

**Affiliations:** 1 Health Services Research, Huntsman Cancer Institute, University of Utah School of Medicine, Salt Lake City, UT 84112, USA

**Keywords:** Adherence, Placebo, Risk factors, Confounding, Observational studies

## Abstract

**Background:**

Previous studies found an association of greater adherence to placebo medication with better outcomes. The present study tested whether this association was explained by any of the following factors: 1) adherence to other medications, 2) healthcare behaviors, 3) disease risk, or 4) predicted degree of adherence. Data included information on more than 800 risk factors from 27,347 subjects in two randomized controlled trials of hormone therapy in the Women's Health Initiative.

**Results:**

Greater adherence to placebo was not associated with colon cancer but was substantially and significantly associated with several diverse outcomes: death, myocardial infarction, stroke, and breast cancer. Adherence to hormone therapy was only weakly associated with outcomes. The WHI risk factors only poorly predicted degree of adherence, R^2^ < 4%. No underlying factors accounted for the association between placebo adherence and outcome.

**Conclusion:**

The results suggest that adherence to placebo is a marker for important risk factors that were not measured by WHI. Once identified these risk factors may be used to increase the validity of observational studies of medical treatment by reducing unmeasured confounding.

## Background

Greater adherence to placebo is associated with lower rates of cardiovascular disease and mortality [1-5]. It has been suggested [6,7] that this association is caused by factors confounded with placebo adherence such as better adherence to active medications or preventive services [8,9], less comorbidity and better functional status [10], and decreasing likelihood of risky behaviors [11]. However, the importance of these factors as an explanation of the healthy adherer effect has not been demonstrated.

The present study evaluates whether the association of outcome and adherence is due to factors commonly measured in epidemiological studies or whether it is necessary to identify new risk factors that influence both adherence and outcomes. For this study we use the comprehensive dataset obtained by the Women’s Health Initiative (WHI). We searched for factors that predict adherence and might be confounded with the association of adherence with outcomes.

## Methods

Several analytic approaches were used to assess the association between adherence and disease: 1) Adherence was tested for an association with several outcomes. If adherence has a much stronger association with cardiovascular diseases than cancers, it would suggest that adherence is associated with behaviors that influence cardiovascular disease more than cancer. 2) Associations between adherence and outcome were compared for women taking medications and women not taking these medications. Stronger associations for women taking medications might imply that adherence to placebo is a marker for greater adherence to other medications that influence outcome. 3) Factors influencing adherence to placebo were compared to factors influencing adherence to hormone therapy. If the same factors influence both adherence to placebo and adherence to hormones, then these same factors are likely to influence adherence to other medications. This is another way of evaluating whether adherence to placebo is a marker for adherence to other medications. 4) We screened many risk factors for health and healthcare behaviors to assess whether any accounted for the association between adherence and outcomes. If this association is substantially reduced after adjustment for certain risk factors, then the association might be mediated by risk factors related to those used in the adjustment.

The Women's Health Initiative study design has been described in detail [12-14]. In brief, it was a long-term national health study that focused on strategies for preventing heart disease, breast and colorectal cancer, and osteoporosis in postmenopausal women. Women between the ages of 50 and 79 were enrolled for an observational study or randomized controlled trial (RCT) from 1993 to 1998 at 40 clinical centers throughout the United States. All participants signed informed consent forms. The institutional review boards at all participating institutions approved the study protocols and procedures. The median follow-up time was eight years. The present study included participants in RCTs of hormone therapy: 16 608 from the RCT of estrogen plus progesterone and 10 739 from the RCT of estrogen only.

### Data

For outcome ascertainment all subjects completed semiannually self-administered, self-reports. Medical records were reviewed for patients who died in the hospital, and autopsy reports were reviewed for patients who had an autopsy. Only the death certificates were reviewed for patients who died outside of the hospital without an autopsy. Outcomes during the follow-up period that were evaluated in the present study included myocardial infarction, stroke, breast cancer, colon cancer, and death. These outcomes were relatively common and sufficiently diverse to show how the association of outcome with adherence varies according to outcome.

The primary risk factor of interest was adherence measured at the time of the one-year follow-up questionnaire. We chose this as our adherence measure because subsequent assessments of adherence might be more weakly associated with the subject’s baseline characteristics and were more likely to be influenced by outcomes that occurred after baseline. The proportion adherence was computed by the WHI as the number of days for which the study medication was dispensed minus the number of days of untaken pills divided by the number of days between visits. The primary adherence score was on a four point scale: 1) ≤80%, 2) >80% to ≤90%, 3) >90% to ≤ 100%, and 4) >100%. Subjects with adherence greater than 100% were those whose pill count was less than the difference between the number of pills provided and number prescribed. These subjects may have lost pills, inadvertently taken extra pills because of an inadequate system for tracking pills, or intentionally took extra pills to gain a hoped for benefit. Lost or extra pills of other subjects must have been less than the missed doses for these subjects.

### Risk factors for outcome

Prior to beginning the present study forward and backward stepwise Cox proportional hazard regression was used to select baseline risk factors for each of the outcomes examined. More than 800 risk factors were candidates for selection in an analysis of outcomes from more than 150,000 women in the WHI. The risk factors for each outcome were as follows:

Myocardial infarction: age, race, income, general health, ability to climb stairs, current smoking, cardiovascular disease, systolic blood pressure, family history of myocardial infarction, treatment for hypertension, history of myocardial infarction, treatment for diabetes, prior coronary artery bypass surgery, and waist-hip ratio.

Stroke: age, race, income, smoking, physical function, systolic blood pressure, cardiovascular disease, diabetes, history of stroke, history of transient ischemic attacks, and treatment for hypertension.

Breast cancer: age, race, history of breast cancer in first degree relative, needle aspirations of the breast, breast biopsy, history of hormone usage, and current hormone use.

Colon cancer: age, waist, history of hormone use, smoking, diabetes, and family history of colon cancer.

Death: age, general health, treatment for diabetes, history of coronary artery bypass surgery, lack of appetite, physical function, systolic blood pressure, and years smoked.

None of the numerous psychosocial measures available from the WHI such as those related to life events, emotional wellbeing, depression, or interpersonal relationships, were independent risk factors for any of the outcomes considered.

#### Statistical methods

One analysis examined the association of adherence with binary variables for healthcare behaviors and education. The strength of this association was measured by comparing the percentage of women who had adherence rates greater than 90% for the two levels of the binary variable. Significance testing of the association was performed using a t test with adherence on a continuous scale.

A second analysis used stepwise linear regression to identify risk factors independently associated with adherence at p<0.001. More than 800 risk factors were candidates for inclusion in the regression equation. Variables were retained in a regression equation only if the statistical significance of the variable was not substantially reduced by using the rank of the values of the variable instead of the raw value (i.e., the association with adherence did not depend on extreme values of the variable). Using these stringent criteria for significance for inclusion in stepwise regression models protected somewhat against including variables with a spurious association with adherence. However, there was no adjustment for multiple comparisons because we wanted to include any characteristics that could possibly be meaningful. This approach has been suggested by a leading epidemiologist [15].

It is possible that the factors that influence adherence to placebo differ from the factors that influence adherence to hormone therapy. To test this possibility, we added to the regression equation an interaction term of each factor with hormone therapy. This analysis was performed in a dataset that combined both hormone therapy and placebo subjects.

The Cox model was used to test whether the level of adherence was a risk factor for a given outcome after adjusting for all of the independent baseline risk factors for that outcome. For these analyses adherence was in four categories and represented by three indicator variables, one for each of the categories except for the highest level of adherence. Therefore, the hazard ratio associated with each level compared the risk at that level to the risk for women at the highest level of adherence. These risk-adjusted hazard ratios for level of adherence were presented in one figure for the subjects taking placebo and one for the subjects taking hormone therapy.

As a measure of a subjects overall risk for a given outcome we used the sum of the products of the values of the patient’s risk factors and the respective regression coefficients in the Cox model for that risk factor. Correlation was used to test the association between adherence and subject risk.

All statistical analyses were performed using SAS version 9 (SAS Institute Inc, Cary, NC).

## Results

As shown in Table 1 most women were ages 56 to 69, Caucasian, had more than a high school education, and had private insurance. Sixty-nine percent of the subjects had adherence rates greater than 90% and 10% had rates greater than 100%. Of the outcomes evaluated the most common was death and the least common was colon cancer.

**Table 1:** Demographic characteristics and outcomes in the study sample

**Characteristics**	**Categories**	**N**	**% of Total**
Age (yrs)	49 to 55	4510	16.52
	56 to 69	16,643	60.96
	70 to 81	6147	22.52
Race	Caucasian	21,997	80.58
	Non-Caucasian	5303	19.42
Education level	No post high school	7994	29.28
	Post high school	11,020	40.37
	College graduate	8286	30.35
Income	< $35,000	13,348	48.89
	35,000 to 75,000	9479	34.72
	≥ 75,000	1467	10.77
	Don’t know	1533	5.62
Insurance type			
(could have more than 1 type)	Private insurance	21,379	78.31
	Medicare	10,157	37.21
	Medicaid	474	1.74
	Other	2017	7.39
	None	2592	9.49
Study			
	RCT of estrogen plus progesterone	16,608	60.73
	RCT of estrogen alone	10,739	39.27
Perecent adherence			
	≤ 50%	2064	7.6%
	> 50% to ≤ 80%	3090	11.4%
	> 80% to ≤ 90%	3195	11.76
	> 90% to ≤ 100%	16,024	58.97
	> 100%	2792	10.27
Outcomes			
	Myocardial infarction	950	3.49
	Stroke	694	2.55
	Breast cancer	941	3.44
	Colon cancer	258	0.94
	Death	1645	6.02

### Predictors of adherence

Table 2 shows the association of adherence with healthcare behaviors and education, which is often associated with healthcare behaviors. Subjects are divided into placebo and hormone therapy arms. The percentage of subjects with adherence rates greater than 90% was lower for women in the hormone therapy arm of the study (66.5%) than the placebo arm (72.0%), P<0.0001. In both study arms the women who engaged in any of the healthcare behaviors listed had a higher adherence rate than women who didn’t. For women taking placebo three healthcare behaviors (taking multivitamins, not smoking, and greater recreational physical activity) were associated with adherence at P<0.0001. For women on hormone therapy taking multivitamins was the only healthcare behavior associated with higher adherence at the P<0.0001 level.

**Table 2:** The percentage of subjects with adherence greater than 90% for subjects with a characteristic and subjects without that characteristic

	**Placebo**				**Hormone therapy**			
**Adherence greater than 90%**	**72.0% (n=13,417)**				**66.5% (n=13,709)**			
**Characteristic**	**Has characteristic (n)**		**t-value**^**‡**^	**P value**	**Has characteristic (n)**		**t-value**^**‡**^	**P value**
	**Yes**^**†**^	**No**^**†**^			**Yes**^**†**^	**No**^**†**^		
Education after high school	71.5% (5360)	72.5% (7958)	−1.32	0.19	66.1% (5588)	66.9% (8024)	−0.94	0.34
Current smoker	65.9% (1391)	72.7% (12,026)	−4.64	<.0001	63.2% (1395)	66.9% (12,314)	−2.20	0.03
Mammogram ever	72.3% (12,518)	68.6% (899)	1.48	0.14	66.8% (12742)	62.6% (976)	2.05	0.04
Colonoscopy ever	73.2% (5340)	71.2% (8077)	1.01	0.31	67.2% (5296)	66.1% (8413)	0.24	0.81
Low fat diet	73.6% (4923)	71.1% (8494)	2.36	0.02	67.1% (5047)	66.1% (8662)	0.56	0.58
Multivitamins	75.2% (4150)	70.6% (9267)	4.84	<.0001	69.4% (4225)	65.2% (9484)	4.30	<.0001
Regular recreational physical activity	73.2% (10,018)	68.6% (3399)	4.39	<.0001	67.3% (9966)	64.4% (3743)	2.41	0.02

‡ A two-sample t-test was used to determine the statistical significance of differences in the adherence scale between subjects with the characteristic and subjects without the characteristic.

† Yes indicates that the participant has the characteristic. No indicates that the participant does not have the characteristic.

The factors independently associated with adherence as determined by linear regression are shown in Table 3. In this table subjects receiving either estrogen alone or estrogen plus progesterone were combined into one hormone therapy group because the specific type of hormone therapy received did not influence adherence. For both subjects given placebo and subjects given hormone therapy the coefficient of determination, R^2^, is very low (less than 4%) indicating that the adherence score was not well predicted by the factors in the model.

**Table 3:** Patient characteristics independently associated with adherence at the P<0.001 level for all subjects

**Variable label**	**All subjects R**^**2**^**=0.036**		**Placebo arm R**^**2**^**=0.035**		**Hormone arm R**^**2**^**=0.036**	
	**t-value**	**P-value**	**t-value**	**P-value**	**t-value**	**P-value**
**Demographic**						
White	14.56	<.0001	10.88	<.0001	9.89	<.0001
Age	−5.28	<.0001	0.89	0.37	−8.01	<.0001
Partner currently retired	4.61	<.0001	4.57	<.0001	1.78	0.075
**Psychological**						
Emotional well being	4.83	<.0001	2.98	0.0029	3.84	0.0001
Wake up several times	4.58	<.0001	2.87	0.0042	3.65	0.0003
Fewer emotional limitations	4.48	<.0001	2.37	0.018	3.84	0.0001
Money problems	−4.08	<.0001	−3.78	0.0002	−2.22	0.027
Attend clubs/lodges/groups	4.05	<.0001	2.95	0.0032	2.78	0.0055
**Healthcare behaviors**						
Interested in dietary modification study	−5.36	<.0001	−4.65	<.0001	−3.01	0.0026
Water Soluble Dietary Fiber (g)	4.20	<.0001	2.66	0.0077	3.10	0.0019
**Medical treatments**						
Nonsteroidal anti-inflammatory	5.08	<.0001	2.41	0.016	4.44	<.0001
Diuretic	4.85	<.0001	3.68	0.0002	3.26	0.0011
Baseline hormone therapy	4.73	<.0001	−0.03	0.97	6.54	<.0001
Any combination pill	4.10	<.0001	3.12	0.0018	2.55	0.011
Years oral contraception	4.08	<.0001	3.43	0.0006	2.49	0.013
**Health**						
Atrial fibrillation	−3.66	0.0003	−1.69	0.091	−3.40	0.0007
Constipation	−3.64	0.0003	−2.33	0.020	−2.86	0.0042
Diastolic blood pressure	−3.37	0.0007	−0.63	0.53	−4.29	<.0001

The associated t-values and P-values are from linear regression analyses.

The table is structured so that the factors are divided into categories and sorted within category by t value from the regression in the dataset with all subjects combined. The most statistically significant factor in both the placebo and hormone therapy arms of the study was race. Greater age was strongly associated with lower adherence for women taking hormone therapy and was not associated with adherence for women taking placebo; the differences between study arms for this association was significant at the P<0.0001 level using a statistical test for interaction.

In general, factors that reflected emotional well-being or contributed to emotional well-being (lack of money problems and participating in groups) were associated with greater adherence. However, adherence was higher for women who woke frequently during the night, possibly because these women believed that hormone therapy might reduce this problem.

Women in both study populations had lower adherence rate if they wanted to participate in the dietary trial and higher adherence rates if they were taking certain types of medications: nonsteroidal anti-inflammatory, diuretics, any combination pill, and more years of oral contraceptives. Taking hormone therapy at baseline, however, had a different association with adherence for women in the placebo group (no association) than for women in the hormone therapy group (it substantially increased adherence). The difference is these associations was significant at the P<0.0001 level using the test for interaction. Women with certain health problems (atrial fibrillation, constipation, and higher diastolic blood pressure) had lower adherence rates.

#### Association of adherence with risk

The results in Table 2 and 3 suggest that adherence is associated with several factors likely to reduce the risk of adverse outcomes, e.g., better healthcare behaviors and better emotional well-being. To examine whether there was a tendency for adherence to be associated with lower risk, we found the correlations between adherence and patient risk for subjects taking placebo). For all outcomes the risk increased with greater adherence. Greater adherence had a correlation of 0.051 (P<0.0001) with breast cancer risk, a correlation of 0.031 (P=0.003) with colon cancer risk, a correlation of 0.022 (P=0.013) with the risk of myocardial infarction, a correlation of 0.017 (P=0.047) with the risk of stroke, and a correlation of 0.008 (P=0.36) with the risk of death.

#### Association of adherence with outcomes

**Figure 1:**
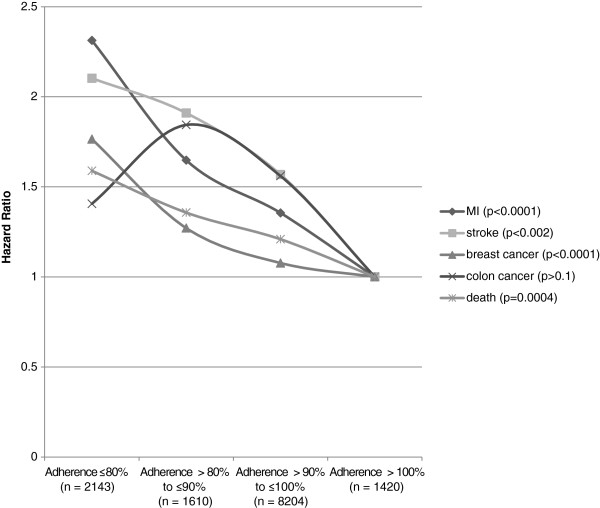
Hazard ratios of placebo adherence for each of five outcomes.

The risk-adjusted association of outcomes with adherence on a four point scale is shown in Figure 1 for subjects given placebo. The group with adherence no greater than 50% was combined with the group that had adherence less than 80% because both groups were small and the outcomes for the group with adherence ≤50% were not worse than other subjects with adherence less than 80%. As shown in the figure the hazard ratios for the two lowest adherence groups were most elevated for myocardial infarction and stroke. The level of statistical significance was also high for breast cancer and death. The association was not statistically significant for colon cancer, which had many fewer cases than the other outcomes.

The association became stronger after adjusting for the risk factors. This was expected because of the positive correlations between adherence and risk. The chi-squared values of adherence in the Cox model before and after adjusting for risk factors were 21.2 and 27.0 for myocardial infarction, 7.8 and 9.5 for stroke, 14.2 and 17.4 for breast cancer, and 11.4 and 12.7 for death. Examples of the adjusted and unadjusted hazard ratios for adherence less than or equal to 80% versus greater than 100% are 2.04 versus 2.31 for myocardial infarction and 1.71 and 1.77 for breast cancer.

Although none of the individual risk factors that predicted adherence were independent risk factors for any outcome, we tested whether the predicted adherence from the linear regression model was a risk factor for MI. After adjusting for other MI risk factors but not adjusting for actual adherence level, the p-value for predicted adherence was 0.82.

One possible explanation of the association of greater adherence to placebo with better health outcomes is that patients who adhere better to placebo also adhere better to other medications. To test these association we tested whether adherence to placebo was more associated with better outcomes for patients taking a particular medication than it was for women not taking the medication, i.e., we tested the statistical significance of the interaction between adherence to placebo and taking a particular type of medication. The medication therapeutic classes evaluated and the number of women in the placebo group who were taking these medications were anti-inflammatory analgesic (n= 2675), diuretic (n= 1627), thyroid (n= 1458), antihypertensive (n= 1447), calcium blocker (n= 1343), antihyperlipedemic (n= 1123), beta_blocker (n= 909), antidiabetic (n= 662), hypnotic (n= 403), antianxiety (n= 357), narcotic analgesic (n= 277), antianginal (n= 195), cardiotonic (n= 156), and anticonvulsant (n= 120). No other types of medication were taken by more than 100 women in the placebo group. Although we tested the interaction of adherence with each of the 14 medication types with 5 outcomes (70 tests in all), none of the tests of these interactions even approached statistical significance at the P<0.10 level. This suggests that the advantage of higher adherence to placebo was not due to greater adherence to any specific, effective, commonly taken medication.

**Figure 2:**
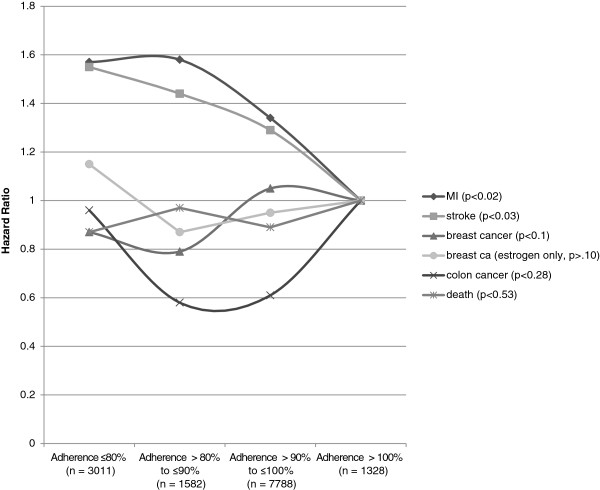
Hazard ratios of hormone therapy adherence for each of five outcomes.

In Figure 2 there is some evidence for declining rates of myocardial infarction and stroke as adherence increased in the subjects on hormone therapy. It is possible that the phenomenon of adherence outweighs any biological risk imparted from the use of hormone therapy? The opposite was observed in the CAST study, in which higher adherence to active therapy was associated with more adverse outcomes [16].

However, associations between adherence and outcome were weaker for subjects on hormone therapy than for subjects on placebo. Based on tests for interaction the association for subjects on placebo was significantly stronger than the association for subjects on hormone therapy for myocardial infarction (P=0.02), breast cancer (P<0.0001), and death (P=0.001). There was little difference in the association between adherence and breast cancer for women on either type of hormone therapy and for women only taking estrogen alone.

## Discussion

Greater adherence to placebo had a substantial and statistically significant association with lower risk for myocardial infarction, stroke, breast cancer, and mortality. Greater adherence to hormone therapy had a weak association with two outcomes (myocardial infarction and stroke) and no significant association with others. The more than 800 risk factors in the WHI only poorly predicted adherence to placebo or hormone therapy and did not account for the associations between adherence and outcome. There was no evidence that the association between placebo adherence and outcome was stronger for patients taking any of the prescribed medications most commonly used by the women in the study. A stronger association for some medications would be expected if placebo adherence was a marker for adherence to this medication.

The association between adherence and outcome is probably due to confounding factors rather than an actual effect of adherence. Our results suggest that these confounding factors were not adequately measured by the WHI. Since WHI is an unusually comprehensive dataset, it is likely that few if any studies record information that accounts for the association between adherence and outcome.

### Previous literature

Previous studies have also found an association of greater adherence to placebo with better healthcare outcomes. Several of these studies have found better cardiovascular and mortality outcomes [1-5], and one even found better outcomes for motor vehicle accidents and workplace accidents [11]. Outcomes not found to be associated with adherence include wrist, vertebral, or non vertebral fractures [17,18].

One study analyzed the same placebo subjects as in the present study [19]. This study found that women with adherence to placebo less than 80% had significantly worse outcomes than women with greater adherence. The hazard ratios were 2.0 for hip fracture, 1.67 for cancer death, 1.56 for all-cause mortality, and 1.45 for myocardial infarction. The following design differences between their study and ours may have contributed to some differences (although no contradictions) in results: 1) Their measure of adherence was based on all follow-up exams and ours was based only on adherence during the first year. As a result the previous study had a higher percentage of subjects with adherence to placebo less than 80% (23% versus 16% in our study). The overall adherence may have been more subject to aspects of health that changed over time and therefore, were not adequately measured at baseline. 2) Their assessment of outcomes was truncated sooner, and they had fewer events: e.g., (241 versus 463 for myocardial infarction), (265 versus 441 for breast cancer) and (464 versus 781 for death). 3) Their study analyzed adherence in two groups and ours in four. 4) The previous study adjusted for 32 pre-specified risk factors in evaluating the association between adherence and outcome. Our study empirically screened more than 800 risk factors to determine if any accounted for the association between adherence and outcome (Additional file 1). 5) The previous study did not examine the association between adherence to hormone therapy and outcome. We are not aware of other studies that examined the association between adherence to an active medication and an outcome that the active medication was not designed to promote. Our results suggest that these associations may differ from the association between placebo and outcome.

## Conclusions

Several studies have found associations of adherence with various diseases and mortality. This probably indicates that adherence to placebo is a marker for other risk factors that influence outcome. However, an exhaustive examination of the comprehensive set of risk factors in the WHI could not identify risk factors that underlie the association between placebo adherence and several outcomes. Because WHI collected a high percentage of the risk factors commonly used to predict cardiovascular diseases and other outcomes, it is also unlikely that many other studies collect these risk factors. The same risk factors that confound the effects of adherence may also confound the effects of medical treatments. If these risk factors are identified, it might increase the validity of observational studies of medical treatments.

## Abbreviations

WHI: Women's Health Initiative; RCT: Randomized controlled trial.

## Competing interests

The authors declare that they have no competing interests.

## Authors’ contribution

AH was responsible for designing the study and drafting the manuscript. TH did the statistical programming and helped with manuscript revisions. Both authors read and approved the final manuscript.

## Supplementary Material

Additional file 1 **Total 971 variables.** Form 2 Eligibility Screening (45). Click here for file
