# Supplementary material for: Why is greater medication adherence associated with better outcomes
Source: Emerg Themes Epidemiol. 2013 Feb 2;10:1. doi: 10.1186/1742-7622-10-1 (PMC3605162; doi:10.1186/1742-7622-10-1)
Supplement: Additional file 1 — Total 971 variables. Form 2 Eligibility Screening (45). [file 1742-7622-10-1-S1.docx]

**Total 971 variables**

**Form 2 Eligibility Screening (45)**

| **Variable** | **Label** |
| --- | --- |
| **AGEHYST** | Hysterectomy age group |
| **AVAILDM** | Available for regular dietary meetings |
| **AVAILHRT** | Consider taking only HRT from CC |
| **BLDPROB** | Bleeding problem ever |
| **CHF_F2** | Heart failure ever |
| **COMECC** | Able to come to clinic |
| **DBDIETF2** | Special diet for diabetes |
| **DIAB** | Diabetes ever |
| **DIABAGE** | Age first told had diabetes |
| **DIABCOMA** | Hospitalized for a diabetic coma |
| **DIABNW** | Diabetes now |
| **DIABPILL** | Pills for diabetes ever |
| **DIABTRT** | Diabetes treated (pills or shots) |
| **DIALYSIS** | Kidney dialysis for kidney failure |
| **DVT_F2** | DVT ever |
| **HARDSTDY** | Problems make it hard to participate |
| **HELPCC** | Kind of help needed to come to clinic |
| **HELPFILL** | Need someone to help fill out forms |
| **HORM** | Female hormones ever |
| **HORM3M** | Female hormones last 3 months |
| **HORMBK** | Hormones to treat osteoporosis fracture |
| **HORMSTAT** | HRT use ever |
| **HRTINFDR** | Send HRT info to Doctor |
| **HYST_F2** | Hysterectomy ever |
| **HYST3M** | Hysterectomy last 3 months |
| **HYSTAGE** | Age at hysterectomy |
| **INSULIN** | Insulin shots ever |
| **INSULINW** | Insulin shots now |
| **INTDM** | Interested in DM part of study |
| **INTHRT_F2** | Interested in HRT part of study |
| **L15LBS6M** | Lost 15 lbs in the last 6 mo w/o trying |
| **LFDIETF2** | Special low-fiber diet |
| **LIVERDIS** | Liver disease ever |
| **MALDIET** | Special malabsorption diet |
| **MEALOUT** | 10 or more meals prepared away from home |
| **MENSELST** | Last time had any menstrual bleeding |
| **MI_F2** | MI ever |
| **MIAGE** | Age first had MI |
| **OSTEOBK** | Osteoporosis-related fracture ever |
| **OTHCHRON** | Other long-term illness |
| **PE_F2** | Pulmonary embolism ever |
| **SCANEMIA** | Sickle cell anemia ever |
| **STROKE_F2** | Stroke ever |
| **TALKDOC** | Interested in talking to Dr. about HRT |
| **TIA_F2** | TIA ever |

**Form 20 Personal Information (47)**

| **Variable** | **Label** |
| --- | --- |
| **ABNPAP3Y** | Abnormal Pap smear last 3 years |
| **ANYINS** | Any Insurance |
| **CAREPROV** | Current Health Care Provider |
| **CERVDYS** | Cervical dysplasia ever |
| **DISABLED** | Currently disabled |
| **EMPLOYED** | Currently employed (full- or part-time) |
| **ENDOASP** | Endometrial aspiration ever |
| **HMOINS** | Pre-paid private insurance |
| **HOMEMKR** | Currently homemaker |
| **INCOME** | Family Income |
| **JOBHMMKR** | Job as homemaker |
| **JOBLABOR** | Job as operator, fabricator, laborer |
| **JOBMANGR** | Job as managerial, professional |
| **JOBOTH** | Job as other than listed |
| **JOBSERV** | Job as service |
| **JOBTECH** | Job as technical, sales, admin support |
| **LSTASPDY** | Days from rand to last aspiration |
| **LSTMAMDY** | Days from rand to last mammogram |
| **LSTPAPDY** | Days from rand to last pap smear |
| **LSTVISDY** | Days from rand to last visit |
| **MAINJOB** | Occupation |
| **MAMMO** | Mammogram ever |
| **MARITAL** | Marital status |
| **MEDICAID** | Medicaid |
| **MEDICARE** | Medicare |
| **MLTRYINS** | Military or VA insurance |
| **NOINS** | No insurance |
| **NOMAM2YR** | No mammogram in last 2 years |
| **NOPAP3YR** | No pap smear in last 3 years |
| **NOTWRK** | Currently not working |
| **OTHPRVIN** | Private insurance (other than pre-paid) |
| **OTHWRK** | Other current job status |
| **PAPSMEAR** | Pap smear ever |
| **PAYOTH** | Other insurance than listed |
| **PDISABLE** | Partner currently disabled |
| **PEDUC** | Partner highest level of education |
| **PEMPLOY** | Partner currently employed |
| **PHOMEMKR** | Partner currently homemaker |
| **PMAINJOB** | Partner's main job |
| **PNOTWRK** | Partner currently not working |
| **POTHWRK** | Partner currently other job |
| **PRETIRED** | Partner currently retired |
| **RETIRED** | Currently retired |
| **TIMELAST** | Time Since Last Medical Visit (months) |
| **TIMELSTS** | Last Medical Visit within 1 Year |
| **USSERVE** | Served in US armed forces |
| **VAMEDCTR** | Used a VA medical center ever |

**Form 30 Medical History (95)**

| **Variable** | **Label** |
| --- | --- |
| **ALS** | ALS ever |
| **ALZHEIM** | Alzheimer's disease ever |
| **ANGINA** | Angina ever |
| **ANGNPILN** | Pills for angina now |
| **AORTICAN** | Aortic aneurysm ever |
| **ARTHRIT** | Arthritis ever |
| **ASTHMA** | Asthma ever |
| **ATRIALFB** | Atrial fibrillation ever |
| **BKBACK** | Broke spine ever |
| **BKBACK55** | Broke spine first time 55 or older |
| **BKBONE** | Broke bone ever |
| **BKFOOT** | Broke foot ever |
| **BKFOOT55** | Broke foot first time 55 or older |
| **BKHAND** | Broke hand ever |
| **BKHAND55** | Broke hand first time 55 or older |
| **BKHIP** | Broke hip ever |
| **BKHIP55** | Broke hip first time 55 or older |
| **BKLARM** | Broke lower arm ever |
| **BKLARM55** | Broke lower arm first time 55 or older |
| **BKLLEG** | Broke lower leg ever |
| **BKLLEG55** | Broke lower leg first time 55 or older |
| **BKOTHB** | Broke other bone ever |
| **BKOTHB55** | Broke other bone first time 55 or older |
| **BKUARM** | Broke upper arm ever |
| **BKUARM55** | Broke upper arm first time 55 or older |
| **BLADCA** | Bladder cancer ever |
| **BRCA_F30** | Breast cancer ever |
| **BRCA55** | Breast cancer 55 or older |
| **CABG** | Coronary bypass surgery ever |
| **CANC_F30** | Cancer ever |
| **CARDCATH** | Cardiac catheterization ever |
| **CARDREST** | Cardiac arrest ever |
| **CAROTID** | Carotid endarterectomy/angioplasty ever |
| **CATARACT** | Cataract ever |
| **CERVCA** | Cervix cancer ever |
| **CHF_F30** | Congestive heart failure ever |
| **COLITIS** | Ulcerative colitis ever |
| **COLN_F30** | Colorectal cancer ever |
| **COLNSCDT** | Date of last colonoscopy |
| **COLNSCPY** | Colonoscopy ever |
| **COLOCA55** | Colorectal cancer 55 or older |
| **CVD** | Cardiovascular disease ever |
| **DIVERTIC** | Diverticulitis ever |
| **EMPHYSEM** | Emphysema ever |
| **FAINTED** | Fainted last 12 months |
| **FRACT55** | Fracture at Age 55+ |
| **GALLBLRM** | Gallbladder removed |
| **GALLBS** | Gallbladder disease or gallstones ever |
| **GALLBSNW** | Gallbladder disease or gallstones now |
| **GALLSTRM** | Gallstones removed |
| **GLAUCOMA** | Glaucoma ever |
| **GOITER** | Goiter ever |
| **GOITERNW** | Goiter now |
| **HEMOCCDT** | Date of last hemoccult test |
| **HEMOCCUL** | Hemoccult test ever |
| **HIBLDCA** | High blood calcium |
| **HICHOLRP** | High cholesterol requiring pills ever |
| **HIP55** | Hip fracture age 55 or older |
| **HIPREP** | Hip replacement ever |
| **HTNTRT** | Hypertension |
| **HYPT** | Hypertension ever |
| **HYPTAGE** | Age told of hypertension |
| **HYPTPILL** | Pills for hypertension ever |
| **HYPTPILN** | Pills for hypertension now |
| **INTESTRM** | Part of intestines removed ever |
| **KIDNEYST** | Kidney or bladder stones ever |
| **LUPUS** | Lupus ever |
| **MELN_F30** | Melanoma cancer ever |
| **MIGRAINE** | Migraine headaches ever |
| **MS** | MS ever |
| **NACOND** | None of listed medical conditions ever |
| **NACVD** | None of the listed CVD conditions ever |
| **NODULE** | Thyroid nodule ever |
| **NODULENW** | Thyroid nodule now |
| **NUMFALLS** | Times fell down last 12 months |
| **OTHERCA** | Other cancers ever |
| **OTHJREP** | Other joint replacement ever |
| **OVRTHY** | Overactive thyroid ever |
| **OVRTHYNW** | Overactive thyroid now |
| **PAD** | Peripheral arterial disease ever |
| **PADANGGR** | Angiography for PAD ever |
| **PADANGP** | Angioplasty for PAD ever |
| **PADSURG** | Surgery to improve flow to legs for PAD |
| **PANCREAT** | Pancreatitis ever |
| **PARKINS** | Parkinson's disease ever |
| **PCOLONRM** | Polyps of colon removed |
| **PTCA** | Angioplasty of coronary arteries ever |
| **REVASC** | CABG/PTCA Ever |
| **RHEUMAT** | Rheumatoid arthritis ever |
| **SKINCA** | Skin cancer (not melanoma) ever |
| **STOMULCR** | Stomach of duodenal ulcer ever |
| **THYRCA55** | Thyroid cancer 55 or older |
| **THYROID** | Thyroid Cancer |
| **UNDTHY** | Underactive thyroid ever |
| **UNDTHYNW** | Underactive thyroid now |

**Form 31 Reproductive History (54)**

| **Variable** | **Label** |
| --- | --- |
| **AGEFBIR** | Age at First Birth |
| **ANYMENSA** | Age at last bleeding |
| **BOOPH** | Bilateral Oophorectomy |
| **BRSTAUG** | Operation to increase breast |
| **BRSTAUGA** | How old at breast augmentation |
| **BRSTBION** | How many breast biopsies |
| **BRSTDIS** | Breast Disease |
| **BRSTFDAF** | How old when first breastfed |
| **BRSTFDAL** | How old when last breastfed |
| **BRSTFDM** | How many months total |
| **BRSTFDMO** | Number of months breastfed |
| **BRSTFDN** | How many children breastfed |
| **BRSTFEED** | Breastfeed at least one month |
| **BRSTIMP** | What type of implant |
| **BRSTOPOT** | Any other breast operations |
| **BRSTPREM** | Removal of part of breast |
| **BRSTREM** | Removal of one or both breasts |
| **BRSTREMO** | Other breast operation |
| **BRTHSTLN** | How many still births |
| **ECTPREG** | How many tubal pregnancies |
| **FULLTRMR** | Full term pregnancy ever |
| **GRAVID** | Number of Pregnancies |
| **MENARCHE** | Age at first period |
| **MENOPSEA** | Age at last regular period |
| **MENPSYAF** | Age at first hot flash |
| **MENPSYAL** | Age at last hot flash |
| **MENPSYMP** | Hot flashes or night sweats |
| **MENSREG** | Were periods regular |
| **MENSREGA** | Age at first regular period |
| **MENSWO1Y** | One year without period |
| **MENSWOD** | Time between first and last period |
| **MISCARYN** | How many miscarriages |
| **NEDLASP** | Needle aspiration ever |
| **NOCNCEIV** | Tried becoming pregnant > 1 yr |
| **NOCNCVDK** | Don't know reason |
| **NOCNCVDR** | Saw doctor because you didn't |
| **NOCNCVEN** | Endometriosis |
| **NOCNCVHR** | Hormones or ovulation |
| **NOCNCVOT** | Other problem with you |
| **NOCNCVPT** | Problem with partner |
| **NOCNCVR** | Reason found for non-pregnancy |
| **NOCNCVUT** | Tubes or uterus |
| **NUMLIVER** | Number of Live Births |
| **OOPH** | One or both ovaries removed |
| **OOPHA** | Age when ovaries removed |
| **PARITY** | Number of Term Pregnancies |
| **PREG** | Ever been pregnant |
| **PREG6M** | Ever have full-term pregnancy |
| **PREG6MAF** | Age at first term pregnancy |
| **PREG6MAL** | Age at last term pregnancy |
| **PREG6MN** | How many times term pregnancy |
| **PREGNUM** | How many times pregnant |
| **TUBTIED** | Ever had tubes tied |
| **TUBTIEDA** | Age when tubes tied |

**Form 32 Family History (79)**

| **Variable** | **Label** |
| --- | --- |
| **BKBCKDAD** | Age father broke spine or back |
| **BKBCKMOM** | Age mother broke spine or back |
| **BKBONDAD** | Father broke a bone after age 40 |
| **BKBONMOM** | Mother broke a bone after age 40 |
| **BKBONREL** | Mom or dad broke bone after age 40 |
| **BKHIPDAD** | Age father broke hip |
| **BKHIPMOM** | Age mother broke hip |
| **BKLARDAD** | Age father broke lower arm |
| **BKLARMOM** | Age mother broke lower arm |
| **BKOTHDAD** | Age father broke other than listed bone |
| **BKOTHMOM** | Age mother broke other than listed bone |
| **BKUARDAD** | Age father broke upper arm |
| **BKUARMOM** | Age mother broke upper arm |
| **BRCADAU1** | Age daughter (1) had breast cancer |
| **BRCADAU2** | Age daughter (2) had breast cancer |
| **BRCADAU3** | Age daughter (3) had breast cancer |
| **BRCAFREL** | Female relative had breast cancer |
| **BRCAGMAM** | Age mat. grandmother had breast cancer |
| **BRCAGMAP** | Age pat. grandmother had breast cancer |
| **BRCAMOM** | Age mother had breast cancer |
| **BRCASIS1** | Age sister (1) had breast cancer |
| **BRCASIS2** | Age sister (2) had breast cancer |
| **BRCASIS3** | Age sister (3) had breast cancer |
| **BRONUM** | Number of brothers |
| **BROTHER** | Have a brother who reached adulthood |
| **CANCFREL** | Female relative had cancer |
| **CANCMREL** | Immediate male blood relative had cancer |
| **CERVREL** | Relative had cervical cancer |
| **CERVRELN** | Number of relatives had cervical cancer |
| **COLOBRO1** | Age brother (1) had colorectal cancer |
| **COLOBRO2** | Age brother (2) had colorectal cancer |
| **COLOBRO3** | Age brother (3) had colorectal cancer |
| **COLODAD** | Age father had colorectal cancer |
| **COLODAU1** | Age daughter (1) had colorectal cancer |
| **COLODAU2** | Age daughter (2) had colorectal cancer |
| **COLOFREL** | Female relative had colorectal cancer |
| **COLOMOM** | Age mother had colorectal cancer |
| **COLOMREL** | Male relative had colorectal cancer |
| **COLOREL** | Male/Female relative had colorectal cancer |
| **COLOSIS1** | Age sister (1) had colorectal cancer |
| **COLOSIS2** | Age sister (2) had colorectal cancer |
| **COLOSIS3** | Age sister (3) had colorectal cancer |
| **COLOSON1** | Age son (1) had colorectal cancer |
| **COLOSON2** | Age son (2) had colorectal cancer |
| **DADAGE** | Natural father's current age |
| **DADALIVE** | Natural father still alive |
| **DADDIEDA** | Age natural father died |
| **DAUGHTER** | Have a daughter who reached adulthood |
| **DAUNUM** | Number of daughters |
| **DIABREL** | Relative had adult diabetes |
| **DIABRELN** | Number of relatives had adult diabetes |
| **ENDOREL** | Relative had endometrial cancer |
| **ENDORELN** | Number of relatives had endomet. cancer |
| **MIBRO1** | Age brother (1) had MI |
| **MIBRO2** | Age brother (2) had MI |
| **MIBRO3** | Age brother (3) had MI |
| **MIDAD** | Age father had MI |
| **MIDAU1** | Age daughter (1) had MI |
| **MIDAU2** | Age daughter (2) had MI |
| **MIMOM** | Age mother had MI |
| **MIREL** | Relatives had heart attack |
| **MISIS1** | Age sister (1) had MI |
| **MISIS2** | Age sister (2) had MI |
| **MISIS3** | Age sister (3) had MI |
| **MISON1** | Age son (1) had MI |
| **MISON2** | Age son (2) had MI |
| **MOMAGE** | Natural mother's current age |
| **MOMALIVE** | Natural mother still alive |
| **MOMDIEDA** | Age natural mother died |
| **OVARREL** | Relative had ovarian cancer |
| **OVARRELN** | Number of relatives had ovarian cancer |
| **PROSREL** | Relative had prostate cancer |
| **PROSRELN** | Number of relatives had prostrate cancer |
| **SISNUM** | Number of sisters |
| **SISTER** | Have a sister who reached adulthood |
| **SON** | Have a son who reached adulthood |
| **SONNUM** | Number of sons |
| **STRKREL** | Relative had a stroke |
| **STRKRELN** | Number of relatives who had a stroke |

**Form 34 Personal Habits (54)**

| **Variable** | **Label** |
| --- | --- |
| **ALC12DR** | Drank 12 alcoholic beverages ever |
| **ALCNOW** | Still drink alcohol |
| **ALCOHOL** | Alcohol intake |
| **ALCQUIT** | Reasons quit drinking alcohol |
| **ALCSWK** | Alcohol servings per week |
| **AVWKEXP** | Energy expend from avg walking |
| **CIGSDAY** | Smoke, cigs/day |
| **COFFEE** | Drink coffee each day |
| **CUPREG** | Number of regular cups of coffee, day |
| **DBDIET34** | Diabetic or ADA diet |
| **FBDIET34** | High-fiber diet |
| **FFWKEXP** | Energy expend fr walking fairly fast |
| **HARDEXP** | Energy expenditure from hard exercise |
| **HRDEX** | Times per week of very hard exercise |
| **HRDEX18** | Very hard exercise 3 times/wk at age 18 |
| **HRDEX35** | Very hard exercise 3 times/wk at age 35 |
| **HRDEX50** | Very hard exercise 3 times/wk at age 50 |
| **HRDEXMIN** | Duration per time of very hard exercise |
| **LACTDIET** | Lactose-free (no milk/dairy foods) diet |
| **LCALDIET** | Low calorie diet |
| **LEPITOT** | Recr. phys activity per week >= 20 Min |
| **LFATDIET** | Low-fat or low cholesterol diet |
| **LMSEPI** | Mod-stren activity >20 min/week (categorical) |
| **LSLTDIET** | Low salt (low sodium) diet |
| **MILDEXP** | Energy expenditure from mild exercise |
| **MLDEX** | Times per week of mild exercise |
| **MLDEXMIN** | Duration per time of mild exercise |
| **MODEX** | Times per week of moderate exercise |
| **MODEXMIN** | Duration per time of moderate exercise |
| **MODEXP** | Energy expend from moderate exercise |
| **MSEPIWK** | Mod. to strenuous phys activity per week |
| **MSMINWK** | Minutes of mod-stren activity per week |
| **OTHDIET** | Other than listed special diet |
| **QSMOKAGE** | Age quit smoking regularly |
| **QSMOKHP** | Quit smoking because of health problems |
| **SEPIWK** | Strenuous activity episodes per week |
| **SMINWK** | Minutes of stren. phys activity per week |
| **SMOKAGE** | Age started smoking cigarettes regularly |
| **SMOKEVR** | Smoked at least 100 cigarettes ever |
| **SMOKING** | Smoking status |
| **SMOKNOW** | Smoke cigarettes now |
| **SMOKWGT** | Smoked to lose weight |
| **SMOKYRS** | Years a regular smoker |
| **TEPIWK** | Recreational phys activity per week |
| **TEXPWK** | Total MET-hours per week |
| **TMINWK** | Minutes of recr. phys activity per week |
| **VFWKEXP** | Energy expend from walking very fast |
| **WALK** | Times walk for > 10 min |
| **WALKEXP** | MET-hours per week from walking |
| **WALKMIN** | Duration of walks when >10 min |
| **WALKSPD** | Walking speed when walking for >10 min |
| **WGTADULT** | Weight during adult life, lbs |
| **XLMSEPI** | Mod-stren activity > 20 min/week |
| **YOYO10LB** | Number times weight went up/down >10 lbs |

**Form 37 Thoughts and Feelings (216)**

| **Variable** | **Label** |
| --- | --- |
| **ACHES** | General aches and pains |
| **ACTDLY** | Activities of Daily Living Construct |
| **AMBEMOT** | Ambivalence over Emotional Expressivenes |
| **ANNOYED** | Becoming easily annoyed or irritable |
| **ANXIOUS** | Feeling nervous, anxious, on edge |
| **APPRVNEG** | Fear others will not approve if negative |
| **BACKSLP** | trouble getting back to sleep |
| **BADLUCK** | Think people make bad luck for sympathy |
| **BADSEX** | People guilty of bad sexual behavior |
| **BATHING** | Bathing or dressing yourself |
| **BENDING** | Bending, kneeling, stooping |
| **BIRD** | Bird |
| **BLOATING** | Bloating or gas |
| **BODPAIN** | How much body pain |
| **BOTHER** | After anger bothered for a long time |
| **CALM** | Felt calm and peaceful |
| **CAREGIV1** | Care Giving Construct #1 (0,1 scoring) |
| **CAREGIV2** | Care Giving Construct #2 (0-5+ scoring) |
| **CAT** | Cat |
| **CGHINCON** | Leak urine when cough, laugh |
| **CHILCON** | Major conflict with children |
| **CLUB** | Attend clubs/lodges/groups last month |
| **CLUMSY** | Clumsiness |
| **COERCE** | Number of people who try to coerce |
| **CONCEN** | Difficulty concentrating |
| **CONSTIP** | Constipation |
| **COUGH** | Coughing or wheezing |
| **COUNTGD** | Rarely count on good things happening |
| **CRYSPELL** | You had crying spells |
| **DIAPER** | Leak protect/Diaper, Attends |
| **DIARRHEA** | Diarrhea |
| **DISAPPNT** | Express disappointment |
| **DIVORCE** | Have a divorce or break-up |
| **DIZZY** | Dizziness |
| **DOG** | Dog |
| **DRESS** | Can you dress and undress self |
| **DWNDUMPS** | Felt down in dumps |
| **EAT** | Can you eat |
| **EMOLIMIT** | Role Limitations Due to Emotional Proble |
| **EMOWELL** | Emotional Well-being |
| **ENERFAT** | Energy/Fatigue |
| **ENERGY** | Had lots of energy |
| **ENJLIF** | You enjoyed life |
| **EXCLUDE** | Number of people who exclude you |
| **EXPCTBST** | Usually expect the best |
| **EXPERTS** | Experts often no better than I |
| **FALLSLP** | fall asleep during quiet activ |
| **FELTBLUE** | Felt downhearted and blue |
| **FELTDEP** | You felt depressed |
| **FELTSAD** | You felt sad |
| **FISH** | Fish |
| **FORGET** | Forgetfulness |
| **FRIENDIE** | Did a close friend die |
| **FRNDIV** | Close friend had a divorce |
| **FRNDSUSE** | Make friends because friends are useful |
| **FRNJOB** | Close friend lost job |
| **FRQINCON** | How often leaked urine |
| **FULLPEP** | Did you feel full of pep |
| **FUN** | Someone to something fun with |
| **GENHEL** | In general, health is |
| **GENHLTH** | General Health Construct |
| **GOODADVC** | Someone to give good advice |
| **GOODTIME** | Someone to have a good time with |
| **HAPPY** | Have you been happy |
| **HEADACHE** | Headaches or migraines |
| **HEARLOSS** | Hearing loss |
| **HEARTBRN** | Heartburn |
| **HEARTRAC** | Heart racing or skipping beats |
| **HLPCHORS** | Someone to help with daily chores |
| **HLPPROB** | Someone to help understand a problem |
| **HLPSICK** | Helping sick family/friend |
| **HLPSICKT** | Times helped sick family/friend |
| **HLTHC1Y** | Compare health to 1 year ago |
| **HLTHEXCL** | My health is excellent |
| **HLTHWORS** | I expect health to get worse |
| **HLTHYANY** | I am as healthy as anybody |
| **HONEST** | Most people are honest due to fear |
| **HOPEFUL** | Always hopeful about future |
| **HOSTIL** | Hostility Construct |
| **HOTFLASH** | Hot flashes |
| **HRSSLP** | How many hours of sleep |
| **HUNGRY** | Increased appetite |
| **INCONDIS** | How much does leakage bother |
| **INCONLMT** | leak limit activities |
| **INCONT** | Ever leaked urine |
| **INOUTBED** | Can you get in and out of bed |
| **INTSOC** | Phys or emotional probs interfere |
| **INTSOC2** | Physical or emotional problem |
| **JNTPAIN** | Joint pain or stiffness |
| **KNWANGRY** | Usually people around know when angry |
| **LEAKAMT** | How much urine you lose |
| **LESSACCE** | Emot/Accomplished less |
| **LESSACCP** | Phys/Accomplished less |
| **LESSCARE** | Emot/Worked less carefully |
| **LESSKNDP** | Phys/limited kind of work |
| **LESSWRKE** | Emot/cut down on time spent |
| **LESSWRKP** | Phys/cut down on time spent |
| **LFEVENT1** | Life Event Construct #1 (0,1 scoring) |
| **LFEVENT2** | Life Event Construct #2 (0-3 scoring) |
| **LIE** | Most people would lie to get ahead |
| **LIFEQUAL** | Rate quality of life |
| **LIFTGROC** | Lifting or carrying groceries |
| **LISTEN** | Someone to listen when need to talk |
| **LIVALN** | Live alone |
| **LIVALOR** | Living Alone |
| **LIVCHLD** | Live with children |
| **LIVFRNDS** | Live with friends |
| **LIVOTH** | Live with other than listed |
| **LIVPRT** | Live with husband/partner |
| **LIVREL** | Live with relatives |
| **LIVSIBL** | Live with brother/sister |
| **LOVE** | Someone to love you/make you feel wanted |
| **LOWBACKP** | Low back pain |
| **MAJACC** | Major accident or disaster |
| **MARRIED** | Currently married or intimate |
| **MEDSLEEP** | take medication for sleep |
| **MENSPAD** | Leak Protection/Menstrual pad |
| **MINIPAD** | Leak Protect/Mini-pad, tissue |
| **MODACT** | Moderate activities |
| **MONPROB** | Major problems with money |
| **MOODSWNG** | Mood swings |
| **MOREGOOD** | Expect more good things than bad |
| **MSCLACHE** | Muscle tension aches or soreness |
| **NAP** | Did you nap during the day |
| **NAUSEA** | Nausea |
| **NECKPAIN** | Neck pain |
| **NEGEMOT** | Negative Emotional Expressiveness (NEE) |
| **NERVES** | Number of people who get on nerves |
| **NERVOUS** | Have you been a very nervous person |
| **NIGHTSWT** | Night sweats |
| **NOCARE** | No one cares what happens to you |
| **NOCONCEN** | Trouble concentrating on things, reading |
| **NOHELP** | People inwardly don't like to help |
| **NOHUNGER** | Decreased appetite |
| **NOINCON** | No longer leak urine |
| **NOPRTCT** | Leak Protect/No protection |
| **NOTMYWAY** | Hardly ever expect things to go my way |
| **OPTIMISM** | Optimism Construct |
| **ORDERS** | Take orders from someone who knew less |
| **OTHINCON** | When leak urine, Other |
| **OTHPET** | Other pet |
| **OTHPRTCT** | Leaking urine protection, Other |
| **PAIN** | Pain Construct |
| **PAININT** | How much did pain interfere |
| **PANIC** | Having an anxiety attack -- feel fear or panic |
| **PEOPDIS** | You felt people disliked you |
| **PET** | Lived with a pet in home |
| **PETDIE** | Did a pet die |
| **PHYAB** | You were physically abused |
| **PHYLIMIT** | Role Limitations Due to Physical Health |
| **PHYSFUN** | Physical Functioning Construct |
| **PSHTDEP** | Shortened CES-D/DIS Screening Instrument |
| **QUALSLP** | Typical night's sleep |
| **RELGTIME** | Times attend religious service/church |
| **RELSTRN** | Religion gives strength and comfort |
| **RESPECT** | People demand more respect than give |
| **RESTLSIT** | Feeling restless so hard to sit still |
| **RESTSLP** | Your sleep was restless |
| **SAD2WK** | Felt sad for two weeks |
| **SAD2YRS** | Felt sad two or more years |
| **SADMUCH** | Felt sad much of past year |
| **SATFRQSX** | Satisfied with sex frequency |
| **SATLIFE** | Satisfied with quality of life |
| **SATSEX** | How satisfied sexually |
| **SCENEPUB** | If angered, cause scene in public place |
| **SEX** | Who you have had sex with |
| **SEX45** | Description of adult sexual orientation |
| **SEXACTIV** | Sexual activity in last year |
| **SEXWORRY** | Sexual activity affect healh |
| **SHARE** | Someone to share private worries/fears |
| **SHOWER** | Can you take a bath or shower |
| **SICKEASY** | I get sick easier |
| **SKINDRY** | Skin dryness or scaling |
| **SLPDSTRB** | Sleep Disturbance Construct |
| **SLPINCON** | Leak when I am sleeping |
| **SNORE** | Did you snore |
| **SOCFUNC** | Social Functioning |
| **SOCSTRN** | Social Strain Construct |
| **SOCSUPP** | Social Support Construct |
| **SPOUSDIE** | Did your spouse die |
| **SPOUSILL** | Did your spouse have a serious illness |
| **STAIR** | Climbing one flight of stairs |
| **STAIRS** | Climbing several flights |
| **STAYSLP** | Trouble falling asleep or staying asleep |
| **SUPPRESS** | Usually suppress anger |
| **SWELLHND** | Swelling of hands or feet |
| **SYMPTOM** | Symptom Construct |
| **TAKEDR** | Someone can take to the doctor |
| **TELLFEEL** | Tell from facial expressions how feeling |
| **TIRED** | Did you feel tired |
| **TIRED2** | Feeling tired |
| **TIREEASY** | Getting tired very easily |
| **TOINCON** | Leak when can't get to toilet |
| **TOOMUCH** | Number of people who ask too much |
| **TRBSEE** | Trouble with vision |
| **TRBSLEEP** | Did you have trouble sleeping |
| **TREMORS** | Tremors |
| **TRUSTNO** | Safer to trust nobody |
| **TRUTH** | Argue to convince people of truth |
| **UNFAIR** | Most people are unfair to gain profit |
| **UPEARLY** | wake up earlier than planned |
| **UPSTOM** | Upset stomach or belly pain |
| **URINPAIN** | Pain/burning while urinating |
| **VAGDIS** | Vaginal or genital discharge |
| **VAGDRY** | Vaginal or genital dryness |
| **VAGITCH** | Vaginal or genital irritation |
| **VERBAB** | You were verbally abused |
| **VIGACT** | Vigorous activities |
| **WAKENGHT** | Did you wake up several times |
| **WALK1BLK** | Walking one block |
| **WALK1M** | Walking more than one mile |
| **WALKBLKS** | Walking several blocks |
| **WELBEING** | Rate current sense of well-being |
| **WORNOUT** | Did you feel worn out |
| **WRKDIFFP** | Phys/difficulty perform work |
| **WRONG** | Expect something that can will go wrong |

**Form 43 Hormone Use (44)**

| **Variable** | **Label** |
| --- | --- |
| **DES** | DES (diethylstilbestrol) use ever |
| **DESAGEMAX** | Age stopped DES |
| **DESAGEMIN** | Age started DES |
| **DESTIME** | DES Duration (years) |
| **DMPA** | DMPA (depo-provera) use ever |
| **DMPAFREQ** | DMPA frequency of use |
| **DMPAGEMAX** | Age last used DMPA |
| **DMPAGEMIN** | Age first used DMPA |
| **DMPATIME** | DMPA duration (years) |
| **DMPAUOM** | DMPA frequency UOM |
| **ESTR** | Estratest use |
| **ESTRMAX** | Age last used estratest |
| **ESTRMIN** | Age first used estratest |
| **ESTRSTAT** | Estratest usage status |
| **ESTRTIME** | Estratest duration |
| **OC** | Oral contraceptive use ever |
| **OCAGEMAX** | Age last used OC |
| **OCAGEMIN** | Age first used OC |
| **OCBPREG** | OC use before first term pregancy |
| **OCBPTIME** | OC use before first term preg duration (years) |
| **OCTIME** | OC duration (years) |
| **PCYCLE** | Progesterone cycle during last PERT use |
| **TEST** | Testosterone or other male hormone use |
| **TESTMAX** | Age last used testosterone |
| **TESTMIN** | Age first used testosterone |
| **TESTSTAT** | Testosterone or other male hormone status |
| **TESTTIME** | Testosterone or other male hormone duratation |
| **TOTE** | Unopposed estrogen use ever |
| **TOTECAT** | Unopposed estrogen duration by category |
| **TOTEMAX** | Age last used unopposed estrogen |
| **TOTEMIN** | Age first used unopposed estrogen |
| **TOTESTAT** | Unopposed estrogen usage status |
| **TOTETIME** | Lifetime unopposed estrogen duration |
| **TOTH** | HRT uuse ever |
| **TOTHCAT** | HRT duration by category |
| **TOTHMAX** | Age last used HRT |
| **TOTHMIN** | Age first used HRT |
| **TOTHSTAT** | HRT usage status |
| **TOTHTIME** | Lifetime HRT duration |
| **TOTP** | Estrogen + progesterone use ever |
| **TOTPMAX** | Age last used estrogen + progesterone |
| **TOTPMIN** | Age first used estrogen + progesterone |
| **TOTPSTAT** | Estrogen + progesterone usage status |
| **TOTPTIME** | Lifetime estrogen + progest duration |

**Form 45 Current Supplements (55)**

| **Variable** | **Label** |
| --- | --- |
| **F45BETA** | Supplemental Beta-carotene, mcg |
| **F45BIOT** | Supplemental Biotin, mcg |
| **F45CALC** | Supplemental Calcium, mg |
| **F45CHROM** | Supplemental Chromium, mcg |
| **F45COMBP** | Any Combination Pill |
| **F45COPP** | Supplemental Copper, mg |
| **F45FOLIC** | Supplemental Folic Acid, mcg |
| **F45IRON** | Supplemental Iron, mg |
| **F45MAGN** | Supplemental Magnesium, mg |
| **F45MANG** | Supplemental Manganese, mg |
| **F45MOLYB** | Supplemental Molybdenum, mcg |
| **F45MULTI** | Multivitamin without Minerals |
| **F45MVMIN** | Multivitamin with Minerals |
| **F45NIAC** | Supplemental Niacin, mg |
| **F45OTHCM** | Other Comb Pill (not multivit/stress) |
| **F45PANTO** | Supplemental Pantothenic Acid, mg |
| **F45PHOS** | Supplemental Phosphorus, mg |
| **F45POTAS** | Supplemental Potassium, mg |
| **F45RETIN** | Supplemental Retinol, mcg |
| **F45SELEN** | Supplemental Selenium, mcg |
| **F45STRES** | Stress Formula Pills |
| **F45VITA** | Supplemental Vitamin A, mcg RE |
| **F45VITB1** | Supplemental Vitamin B1 (Thiamine), mg |
| **F45VITB2** | Supplemental Vitamin B2 (Thiamine), mg |
| **F45VITB6** | Supplemental Vitamin B6, mg |
| **F45VITC** | Supplemental Vitamin C, mg |
| **F45VITD** | Supplemental Vitamin D, mcg |
| **F45VITE** | Supplemental Alpha-tocopherol, IU |
| **F45VTB12** | Supplemental Vitamin B12, mcg |
| **F45ZINC** | Supplemental Zinc, mg |
| **TKBIOT** | F45 Taking biotin from single sup |
| **TKCALC** | F45 Taking calcium from single sup |
| **TKCHROM** | F45 Taking chromium from single sup |
| **TKCOPP** | F45 Taking copper from single sup |
| **TKFOLIC** | F45 Taking folic acid from single sup |
| **TKIRON** | F45 Taking iron from single sup |
| **TKMAGN** | F45 Taking magnesium from single sup |
| **TKMANG** | F45 Taking manganese from single sup |
| **TKMOLYB** | F45 Taking molybdenum from single sup |
| **TKNIAC** | F45 Taking niacin from single sup |
| **TKPANTO** | F45 Taking pantoth. acid from single sup |
| **TKPHOS** | F45 Taking phosphorus from single sup |
| **TKPOTAS** | F45 Taking potassium from single sup |
| **TKRETIN** | F45 Taking retinol from single sup |
| **TKSELEN** | F45 Taking selenium from single sup |
| **TKVITA** | F45 Taking vitamin A from single sup |
| **TKVITB1** | F45 Taking vitamin B1 from single sup |
| **TKVITB12** | F45 Taking vitamin B12 from single sup |
| **TKVITB2** | F45 Taking vitamin B2 from single sup |
| **TKVITB6** | F45 Taking vitamin B6 from single sup |
| **TKVITC** | F45 Taking vitamin C from single sup |
| **TKVITD** | F45 Taking vitamin D from single sup |
| **TKVITE** | F45 Taking alpha-toco from single sup |
| **TKZINC** | F45 Taking zinc from single sup |

**Form 60 Energy (139)**

| **Variable** | **Label** |
| --- | --- |
| **F60ACARO** | Dietary Alpha-Carotene (mcg) |
| **F60ADSGR** | Dietary Added Sugars (g) |
| **F60ALAN** | Dietary Alanine (g) |
| **F60ALC** | Dietary Alcohol (g) |
| **F60ALCWK** | Alcohol servings per week |
| **F60ANMPR** | Dietary Animal Protein (g) |
| **F60ARGIN** | Dietary Arginine (g) |
| **F60ASH** | Dietary Ash (g) |
| **F60ASPRT** | Dietary Aspartic Acid (g) |
| **F60ATOCO** | Dietary Alpha-Tocopherol (mg) |
| **F60BCRYP** | Dietary Beta-Cryptoxanthin (mcg) |
| **F60BETA** | Dietary Beta-Carotene (mcg) |
| **F60BIOCHN** | Dietary Biochanin A (mg) |
| **F60BTOCO** | Dietary Beta-Tocopherol (mg) |
| **F60CAFF** | Dietary Caffeine (mg) |
| **F60CALC** | Dietary Calcium (mg) |
| **F60CARB** | Dietary Total Carbohydrate (g) |
| **F60CBPCT** | Percent Calories from Carbohydrates |
| **F60CHOLS** | Dietary Cholesterol (mg) |
| **F60COPPR** | Dietary Copper (mg) |
| **F60CUMST** | Dietary Coumestrol (mg) |
| **F60CYSTN** | Dietary Cystine (g) |
| **F60DAIDZ** | Dietary Daidzein (mg) |
| **F60DIETGA** | Dietary Glycemic Index (using available carbs) |
| **F60DIETGI** | Dietary Glycemic Index (using total carbs) |
| **F60DTOCO** | Dietary Delta-Tocopherol (mg) |
| **F60ENRGY** | Dietary Energy (kcal) |
| **F60ENRGYJ** | Dietary Energy (joules) |
| **F60FAT** | Dietary Total Fat (g) |
| **F60FIBER** | Dietary Fiber (g) |
| **F60FLDEQ** | Dietary Folate Equivalents (mcg) |
| **F60FLNAT** | Dietary Natural Folate (food folate) (mcg) |
| **F60FLSYN** | Dietary Synthetic Folate (folic acid) (mcg) |
| **F60FOLA** | Dietary Folacin (mcg) |
| **F60FRMNT** | Dietary Formononetin (mg) |
| **F60FRUCT** | Dietary Fructose (g) |
| **F60FRUIT** | Daily Fruit Consumption (med portion) |
| **F60FTPCT** | Percent Calories from Fat |
| **F60GALAC** | Dietary Galactose (g) |
| **F60GLAC** | Dietary Glycemic Load Based on Available Carb |
| **F60GLUC** | Dietary Glucose (g) |
| **F60GLUT** | Dietary Glutamic Acid (g) |
| **F60GLYCN** | Dietary Glycine (g) |
| **F60GLYCTN** | Dietary Glycitein (mg) |
| **F60GNISTN** | Dietary Genistein (mg) |
| **F60GRAMS** | Dietary Gram Amount |
| **F60GTLC** | Dietary Glycemic Load Based on Total Carb |
| **F60GTOCO** | Dietary Gamma-Tocopherol (mg) |
| **F60HISTD** | Dietary Histidine (g) |
| **F60INSFB** | Insoluble Dietary Fiber (g) |
| **F60IRON** | Dietary Iron (mg) |
| **F60ISOLE** | Dietary Isoleucine (g) |
| **F60LACT** | Dietary Lactose (g) |
| **F60LEUCN** | Dietary Leucine (g) |
| **F60LUTZX** | Dietary Lutein+Zeaxanthin (mcg) |
| **F60LYCO** | Dietary Lycopene (mcg) |
| **F60LYSIN** | Dietary Lysine (g) |
| **F60MAGN** | Dietary Magnesium (mg) |
| **F60MALT** | Dietary Maltose (g) |
| **F60MANGN** | Dietary Manganese (mg) |
| **F60METH** | Dietary 3-Methylhistidine (mg) |
| **F60METHN** | Dietary Methionine (g) |
| **F60MF141** | Dietary MFA 14:1 (g) |
| **F60MF161** | Dietary MFA 16:1 (g) |
| **F60MF181** | Dietary MFA 18:1, Oleic Acid (g) |
| **F60MF201** | Dietary MFA 20:1 (g) |
| **F60MF221** | Dietary MFA 22:1 (g) |
| **F60MFA** | Dietary Total MFA (g) |
| **F60MFPCT** | Percent Calories from MFA |
| **F60NATOC** | Dietary Natural Alpha-Tocopherol (mg) |
| **F60NIACN** | Dietary Niacin (mg) |
| **F60NICNEQ** | Dietary Niacin Equivalents (mg) |
| **F60OMGA3** | Dietary Omega 3 (g) |
| **F60OMGA6** | Dietary Omega 6 FA (g) |
| **F60OXALC** | Dietary Oxalic Acid (mg) |
| **F60PANTO** | Dietary Pantothenic Acid (mg) |
| **F60PECT** | Dietary Pectins (g) |
| **F60PF182** | Dietary PFA 18:2, Linoleic Acid (g) |
| **F60PF183** | Dietary PFA 18:3, Linolenic Acid (g) |
| **F60PF184** | Dietary PFA 18:4 (g) |
| **F60PF204** | Dietary PFA 20:4 (g) |
| **F60PF205** | Dietary PFA 20:5, EPA (g) |
| **F60PF225** | Dietary PFA 22:5 (g) |
| **F60PF226** | Dietary PFA 22:6, dha (g) |
| **F60PFA** | Dietary Total PFA (g) |
| **F60PFPCT** | Percent Calories from PFA |
| **F60PHNYL** | Dietary Phenylalanine (g) |
| **F60PHOS** | Dietary Phosphorous (mg) |
| **F60PHYTC** | Dietary Phytic Acid (mg) |
| **F60POTAS** | Dietary Potassium (mg) |
| **F60PROLN** | Dietary Proline (g) |
| **F60PROT** | Dietary Protein (g) |
| **F60PRPCT** | Percent Calories from Protein |
| **F60RETIN** | Dietary Retinol (mcg) |
| **F60RIBO** | Dietary Riboflavin (mg) |
| **F60SELEN** | Dietary Selenium (mcg) |
| **F60SERIN** | Dietary Serine (g) |
| **F60SF100** | Dietary SFA 10:0 (g) |
| **F60SF120** | Dietary SFA 12:0 (g) |
| **F60SF140** | Dietary SFA 14:0 (g) |
| **F60SF160** | Dietary SFA 16:0, Palmitic Acid (g) |
| **F60SF170** | Dietary SFA 17:0 (g) |
| **F60SF180** | Dietary SFA 18:0, Stearic Acid (g) |
| **F60SF200** | Dietary SFA 20:0 (g) |
| **F60SF220** | Dietary SFA 22:0 (g) |
| **F60SF40** | Dietary SFA 4:0 (g) |
| **F60SF60** | Dietary SFA 6:0 (g) |
| **F60SF80** | Dietary SFA 8:0 (g) |
| **F60SFA** | Dietary Total SFA (g) |
| **F60SFPCT** | Percent Calories from SFA |
| **F60SODUM** | Dietary Sodium (mg) |
| **F60SOLFB** | Water Soluble Dietary Fiber (g) |
| **F60STOCO** | Dietary Synthetic Alpha-Tocopherol (mg) |
| **F60STRCH** | Dietary Starch (g) |
| **F60SUCR** | Dietary Sucrose (g) |
| **F60TF161** | Dietary Trans Fatty Acid, 161T (g) |
| **F60TF181** | Dietary Trans Fatty Acid, 181T (g) |
| **F60TF182** | Dietary Trans Fatty Acid, 182T (g) |
| **F60TFTOT** | Dietary Total Trans Fatty Acid (g) |
| **F60THIAM** | Dietary Thiamin (mg) |
| **F60THREO** | Dietary Threonine (g) |
| **F60TRYPT** | Dietary Tryptophan (g) |
| **F60TSUGR** | Dietary Total Sugars (g) |
| **F60TYROS** | Dietary Tyrosine (g) |
| **F60VALIN** | Dietary Valine (g) |
| **F60VB12** | Dietary Vitamin B12 (mcg) |
| **F60VEG** | Daily Vegetable Consumption (med portion) |
| **F60VEGPR** | Dietary Vegetable Protein (g) |
| **F60VITA** | Dietary Vitamin A (RAE) |
| **F60VITAIU** | Dietary Vitamin A (IU) |
| **F60VITARE** | Dietary Vitamin A (mcg RE) |
| **F60VITB6** | Dietary Vitamin B6 (mg) |
| **F60VITC** | Dietary Vitamin C (mg) |
| **F60VITD** | Dietary Vitamin D (mcg) |
| **F60VITE** | Dietary Total Alpha-Toc Eq (mg) |
| **F60VITK** | Dietary Vitamin K (NDS Value) (mcg) |
| **F60VTEIU** | Dietary Vitamin E (IU) |
| **F60WATER** | Dietary Water (g) |
| **F60ZINC** | Dietary Zinc (mg) |

**Form 80 and others (41)**

| **Variable** | **Label** |
| --- | --- |
| **BMICX** | BMI Categorical |
| **BMIX** | BMIX |
| **DIAS** | Diastolic BP |
| **DIASBP1** | Diastolic blood pressure (1st reading) |
| **DIASBP2** | Diastolic blood pressure (2nd reading) |
| **DIASTOL** | Diastolic BP |
| **group21** | Antineoplastics |
| **group24** | Estrogens |
| **group249930** | Estrogen & Progestin |
| **group26** | Progestins |
| **group28** | THYROID |
| **group32** | ANTIANGINAL AGENTS |
| **group33** | BETA BLOCKERS |
| **group34** | CALCIUM BLOCKERS |
| **group35** | ANTIARRHYTHMIC |
| **group36** | ANTIHYPERTENSIVE |
| **group37** | DIURETICS |
| **group38** | PRESSORS |
| **group39** | Antihyperlipidemic |
| **group40** | MISC. CARDIOVASCULAR |
| **group57** | Antianxiety Agents |
| **group58** | Antidepressants |
| **group59** | Antipsychotics |
| **group60** | Hypnotics |
| **group64** | ANALGESICS - NONNARCOTIC |
| **group65** | ANALGESICS - NARCOTIC |
| **group72** | ANTICONVULSANT |
| **HEMATOCR** | Hematocrit (%) |
| **HEIGHTX** | **Height cm** |
| **HIPX** | **Hip circumference cm** |
| **PLATELET** | Platelet count (Kcell/ml) |
| **PULSE30** | Resting pulse in 30 seconds |
| **SYST** | Systolic BP |
| **SYSTBP1** | Systolic blood pressure (1st reading) |
| **SYSTBP2** | Systolic blood pressure (2nd reading) |
| **SYSTOL** | Systolic BP |
| **WAISTX** | **Waist circumference cm** |
| **WBC** | White blood cell (Kcell/ml) |
| **WEIGHTX** | **Weight kg** |
| **WHEXPECT** | Waist and Hip measurement expected |
| **WHRX** | **Waist hip ratio** |
